# Role of germinal center and CD39^high^CD73^+^ B cells in the age-related tonsillar involution

**DOI:** 10.1186/s12979-024-00425-4

**Published:** 2024-04-12

**Authors:** Rocío Pastor, Juliana Puyssegur, M. Paula de la Guardia, Lindybeth Sarmiento Varón, Gladys Beccaglia, Nicolás Spada, Andrea Paes de Lima, M. Soledad Collado, Andrés Blanco, Isabel Aspe Scetti, M. Elena Arabolaza, Bibiana Paoli, Fernando Chirdo, Eloísa Arana

**Affiliations:** 1 Institute of Immunology, Genetics and Metabolism (INIGEM), Clinical Hospital ‘José de San Martín’, University of Buenos Aires (UBA), National Council for Scientific and Technological Research (CONICET), Av Córdoba 2351, C1120AAF, Buenos Aires, CABA Argentina; 2 Department of Biological Sciences, Faculty of Exact Sciences, Institute of Immunological and Physiopathological studies (IIFP), University of La Plata (UNLP), National Council for Scientific and Technological Research (CONICET), La Plata, Argentina; 3 Department of Pathology, Clinical Hospital ‘José de San Martín’, University of Buenos Aires (UBA), Buenos Aires, Argentina; 4 Institute of Otolaryngology Arauz, Buenos Aires, Argentina; 5 Pediatric Otolaryngology Division, Clinical Hospital ‘José de San Martín’, University of Buenos Aires (UBA), Buenos Aires, Argentina; 6 Department of Immunology, School of Medicine, University of Buenos Aires (UBA), Buenos Aires, Argentina

**Keywords:** Tonsils, Mucosa, Immunity, Ageing, Inflammation, Regulation

## Abstract

**Background:**

The tonsils operate as a protection ring of mucosa at the gates of the upper aero-digestive tract. They show similarities with lymph nodes and participate as inductive organs of systemic and mucosal immunity. Based on the reduction of their size since puberty, they are thought to experience involution in adulthood. In this context, we have used tonsillar mononuclear cells (TMC) isolated from patients at different stages of life, to study the effect of ageing and the concomitant persistent inflammation on these immune cells.

**Results:**

We found an age-dependent reduction in the proportion of germinal center B cell population (B_GC_) and its T cell counterpart (T follicular helper germinal center cells, Tfh_GC_). Also, we demonstrated an increment in the percentage of local memory B cells and mantle zone T follicular helper cells (mTfh). Furthermore, younger tonsils rendered higher proportion of proliferative immune cells within the freshly isolated TMC fraction than those from older ones. We demonstrated the accumulation of a B cell subset (CD20^+^CD39^high^CD73^+^ cells) metabolically adapted to catabolize adenosine triphosphate (ATP) as patients get older. To finish, tonsillar B cells from patients at different ages did not show differences in their proliferative response to stimulation ex vivo, in bulk TMC cultures.

**Conclusions:**

This paper sheds light on the changing aspects of the immune cellular landscape, over the course of time and constant exposure, at the entrance of the respiratory and digestive systems. Our findings support the notion that there is a re-modelling of the immune functionality of the excised tonsils over time. They are indicative of a transition from an effector type of immune response, typically oriented to reduce pathogen burden early in life, to the development of an immunosuppressive microenvironment at later stages, when tissue damage control gets critical provided the time passed under immune attack. Noteworthy, when isolated from such histologic microenvironment, older tonsillar B cells seem to level their proliferation capacity with the younger ones. Understanding these features will not only contribute to comprehend the differences in susceptibility to pathogens among children and adults but would also impact on vaccine developments intended to target these relevant mucosal sites.

**Supplementary Information:**

The online version contains supplementary material available at 10.1186/s12979-024-00425-4.

## Background

In humans, the local mucosal immunity in upper airways is secured mostly by the Waldeyer ring (paired palatine tonsils, adenoids and lingual tonsils). Based on the reduction of their size, it has long been assumed that palatine tonsils undergo some kind of involution from puberty onwards [1]. Still, the mechanisms of such partial involution and their impact on immunity are yet to be determined.

Recurrent tonsillitis (RT) and tonsillar hypertrophy (TH) are the two main causes for tonsillectomy in children and teenagers [2]. In fact, TH is the major motive for tonsillectomy in young children (below 10 years old) [3, 4]. Around puberty, a shift occurs from TH towards RT as the primary reason for the indication of surgery [2, 3]. RT is defined as a number of repetitive infections of tonsils per year, characterized by systemic and local symptoms including fever, sore throat and tonsillar exudate. On the other hand, TH has been long considered as non-infectious by ear, nose and throat (ENT) specialists. In this case, the problem is posed by the spatial obstruction caused by the TH.

The temporal pattern from TH to RT as the main cause of surgery at a population level constitutes a fundamental observation underlying our working hypothesis. We have recently shown that TH has infectious nature by evidencing bacterial penetration through the epithelial layer to the lymphoid compartment in TH samples [4]. Thus, the characteristic follicular hyperplasia that defines the TH condition can be attributable to the persistent, sub-clinical infection detected. Our current hypothesis is that the age-related changes of tonsillar immunity combined with the anti-inflammatory mechanisms triggered to avoid immunopathology, reflect on the increased number of tonsillectomies due to RT after puberty.

In this regard, we have demonstrated that TMC from TH exhibited a pro-inflammatory cytokine profile in culture and intensively active germinal centers [4]. Germinal centers in young human tonsils are long lasting, fueling strong effector immune responses.

We have also reported that TH tonsils rendered significantly lower percentages of IL10-producing B cells (Bregs) than tonsils excised due to RT [2]. There are also regulatory mechanisms exerted by B cells which are independent of IL10 like adenosine (ADO)-producing CD39^high^CD73^+^B cells [5]. CD39 and CD73 are ecto-nucleotidases that can hydrolyze extracellular ATP yielding adenosine 5’-monophosphate (5′-AMP) and ADO. Interestingly, human tonsillar cryosections have long been used to study extracellular nucleotide catabolism [6, 7].

In the present article we demonstrate that the proportion of tonsillar B_GC_ and its T cell counterpart, Tfh_GC_, both decrease with increasing age in the samples we work with. Moreover, we show the progressive loss of capacity of the GC in terms of proliferative aptitude (Ki-67 expression). We extend our previous observations by evidencing the accumulation of CD39^high^CD73^+^B cells in samples from older children. Finally, we demonstrate that the aged-related decay in GC proportion is not recapitulated when stimulating TMC ex vivo. These results imply that the appearance of recurrent tonsillar infections as the main cause of tonsillectomy in teenagers correlates with an escalation of local immune regulation and concomitant deterioration of the effector mechanisms. The paper provides a better comprehension of the dynamic forces of the immune response in the course of time at the gateway of the upper airways.

## Results

### Germinal center population steadily decreases with increasing age

Human tonsils were procured from patients aged between 2 and 39 years old (N = 95, Suppl. Fig. 1). The children and teenagers recruited underwent surgeries from 2020 until 2023 (N = 76, Suppl. Fig. 1 and Table 1) at the Clinical Hospital ‘José de San Martín’. Adult samples were obtained from tonsillectomies in 2023 at the Institute of Otolaryngology Arauz (N = 19, Suppl. Fig. 1 and Table 1). The latter were only used to confirm the trend observed from younger to older ages among the pediatric cohort. Being human samples, most of them from children and some of them obtained during pandemic closures, not all of them were subjected to all determinations. A flow chart with the trace of the samples is provided in Suppl. Fig. 1.

**Table 1:** Basic demographic data of tonsils donors

Age group (years)	Gender				Total	
	Males		Females			
	No^a^	%^b^	No^a^	%^b^	No^a^	%^b^
2–4	14	14,74	6	6,31	20	21,05
5–8	15	15,79	16	16,84	31	32,63
9–11	7	7,37	9	9,47	16	16,84
12–18	7	7,37	2	2,1	9	9,47
> 18	13	13,68	6	6,31	19	20
TOTAL	56	58,95	39	41,05	95	100

^a^The values represent the number of patients in each group who donated samples after undergoing surgery.

^b^The percentages were calculated relative to the total number of patients (n = 95)

Initially, we explored age-related functional changes from early childhood up to 18 years old (Table 2). The TMC extracted from those samples were analyzed by flow cytometry and B_GC_ cells were scored at different ages, as a read out of the effector immunological activity of these organs.

**Table 2:** Basic demographic data of tonsils donors for germinal center (GC) and memory B cells (Bmem) analysis

Age group (years)	Gender				Total	
	Males		Females			
	No^a^	%^b^	No^a^	%^b^	No^a^	%^b^
2–4	14	18,42	6	7,89	20	26,31
5–8	15	19,73	16	21,05	31	40,8
9–11	7	9,21	9	11,84	16	21,05
12–18	7	9,21	2	2,63	9	11,84
TOTAL	43	56,57	33	43,41	76	100

^a^The values represent the number of patients in each group who donated samples after undergoing surgery.

^b^The percentages were calculated relative to the total number of patients (n = 76)

**Fig. 1:**
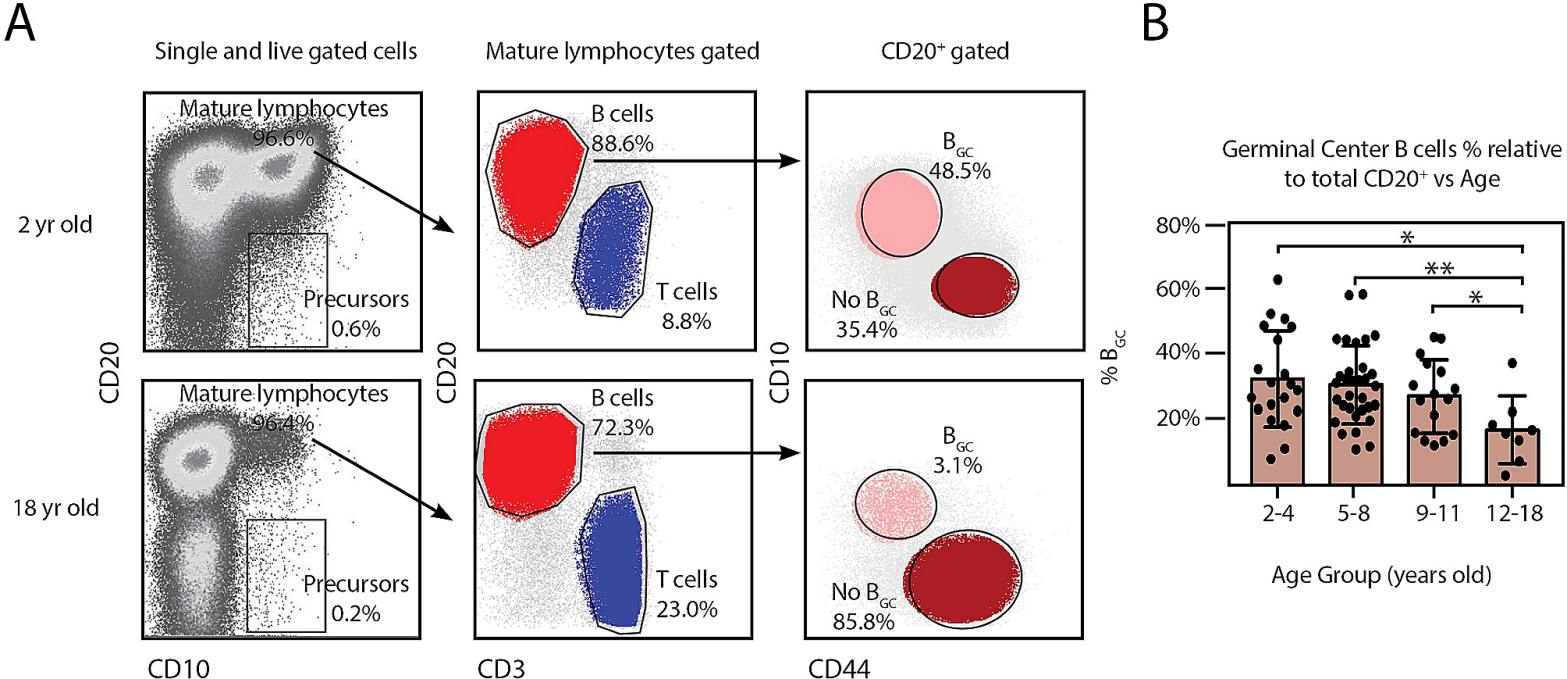
Age dependent distribution of tonsillar B-cell subpopulations. (**A**) Fresh TMC were stained for surface CD20, CD10, CD3, CD44, CD38, CD27 and a dead/live stain as in [8]. Samples were subsequently analyzed by FACS. The gating strategy to identify the different B-cell populations is partially illustrated. Singlets were gated by plotting FSC-H vs. FSC-A for each sample (not shown). Within the singlets population, dead cells were determined by a viability dye (not shown). Within the viable gate, lymphoid gate was determined through SSC-A vs. FSC-A (not shown). Dot plot depicting the representative percentage of the tonsillar B cells subsets for individuals from the youngest (top) and oldest (bottom) groups of patients analyzed. Percentages and colors designate frequencies of the populations indicated. (**B**) Histograms presenting the mean percentage ± SD of the B_GC_ cell population frequencies determined as in A), from 76 individuals, distributed according their age. *p* value was calculated through unpaired *t* test, *p < 0.05; **p < 0.01

**Fig. 2:**
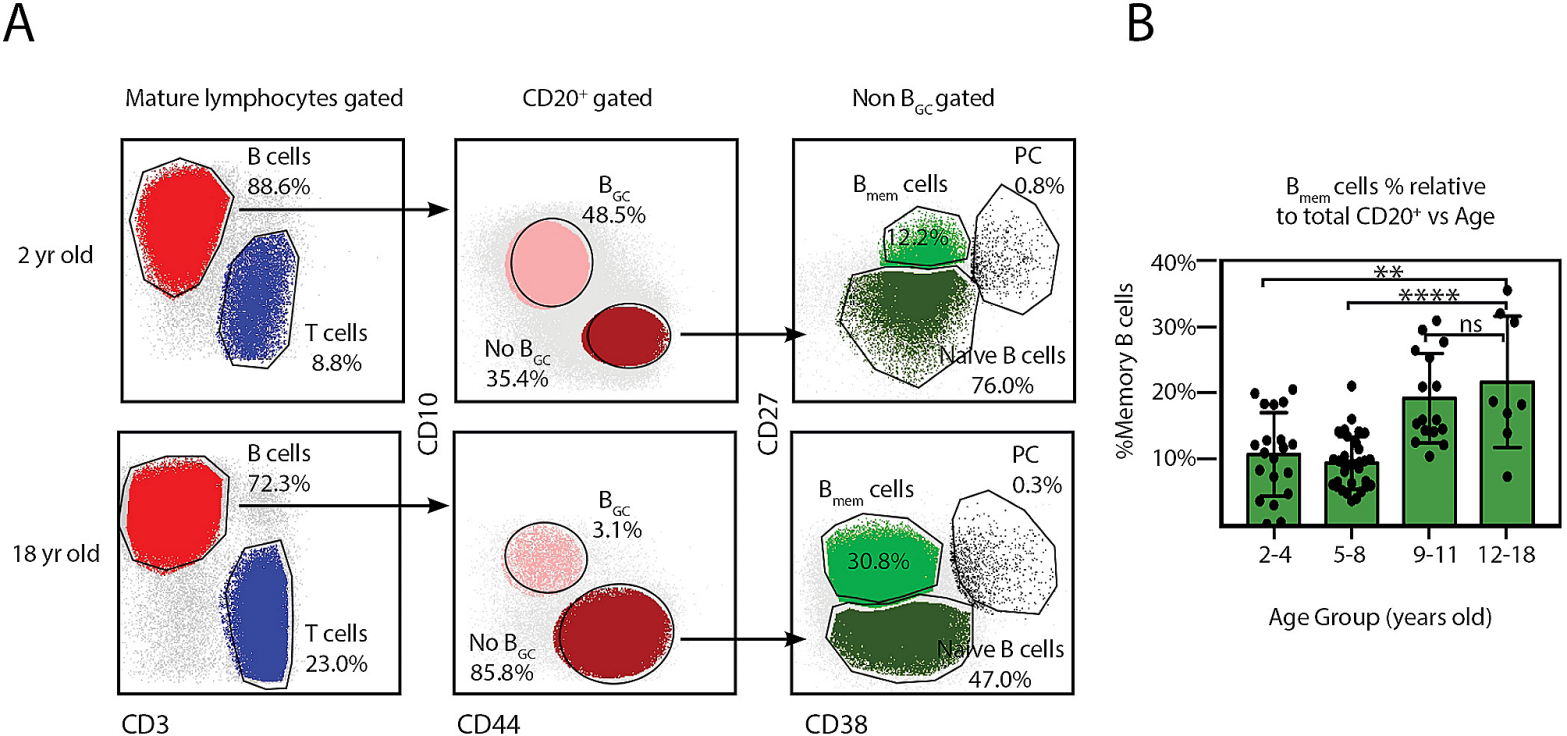
Memory B-cells displace B_GC_ with advancing age. (**A**) Fresh TMC were stained for surface CD20, CD10, CD3, CD44, CD38, CD27 and a dead/live stain as in [8]. Samples were subsequently analyzed by FACS. The gating strategy to identify the different B-cell populations is partially illustrated as in Fig. 1. Percentages and colors designate frequencies of the populations indicated. B_mem_= memory B cells, PC = plasma cells (**B**) Histograms presenting the mean percentage ± SD of the memory B cell population frequencies determined as in A), from 76 individuals, distributed according their age. *p* value was calculated through unpaired *t* test, **p < 0.01; **** p < 0.0001

First, single lymphocytes were gated based on forward and side scatter properties, and tonsillar mature B lymphocytes were identified as CD3^−^CD20^+^CD10^+/−^ cells within the singlets and lymphocyte gate [8]. B_GC_ cells were defined by bright CD10 and low CD44 expression (Fig. 1A). We found that the proportion of B_GC_ cells within CD20^+^ cell population steadily declined with increasing age. B_GC_ cells represented slightly over one third of all the B cells from tonsils within the (2–4) year old range (32.6% ± SD 14.7%). Tonsils from children between 5-8 years old rendered 30.8% ± SD 11.9% B_GC_ cells and in the pre-teens (9–11 years old) group we obtained 27.3% ± SD 11.2% B_GC_. We found a statistically significant lower proportion of B_GC_ (17.0% ± SD 10.3%) in tonsils from teenagers (12–18 years old) when compared to the youngest group (Fig. 1B). In fact, the three younger intervals of age rendered statistically significant higher proportion of B_GC_ when compared with the teenagers group. Moreover, the frequency of memory B cells was significantly lower in the tonsils from the toddlers (2–4 years old) and younger children (5–8 years old) than that of the oldest patients (Fig. 2A and B). Being the GC a crucial reserve of the effector B cell population, we concluded that from a cellular perspective, local effector immune responses appear reduced as children aged. On the other hand, memory B cells accumulated over time.

**Fig. 3:**
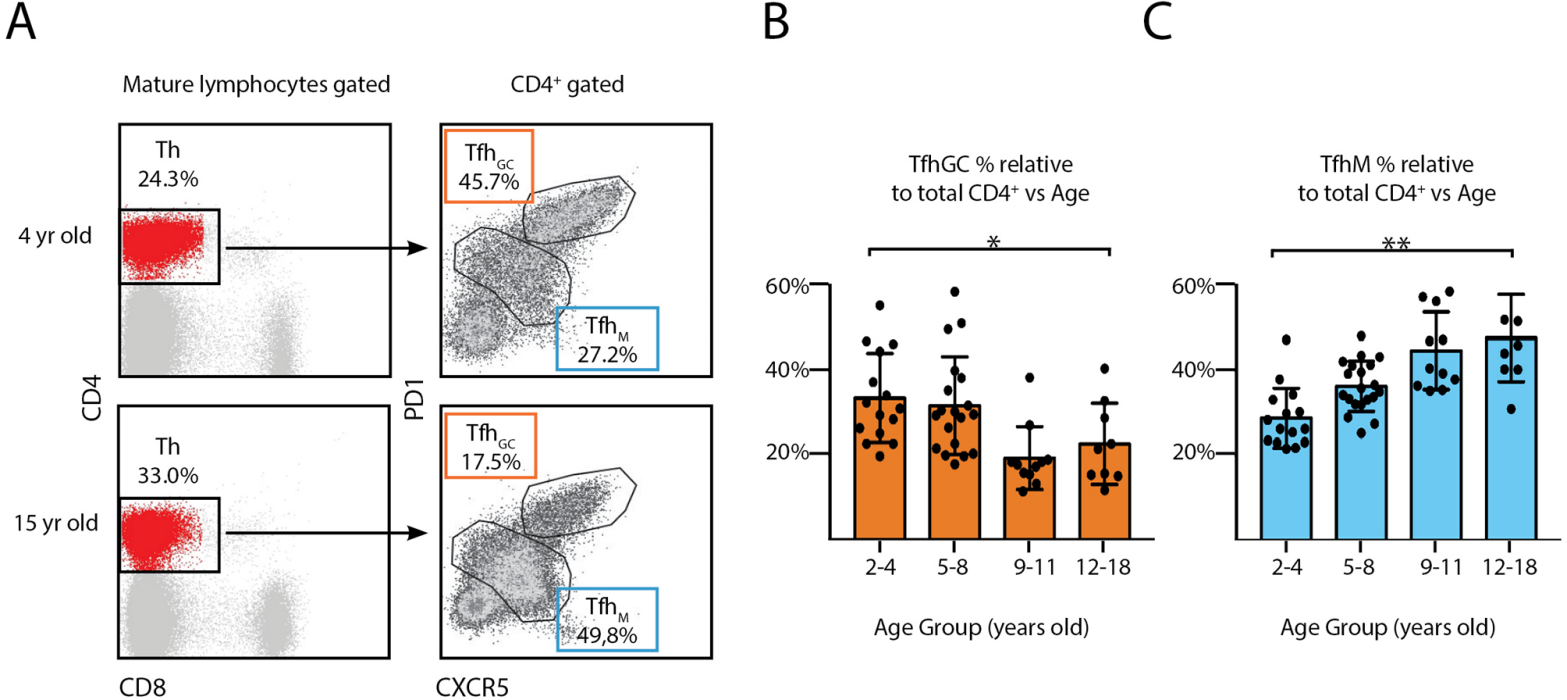
Age dependent distribution of tonsillar follicular T-cell subpopulations. (**A**) Fresh TMC were stained for surface CD4, CD8, PD 1, CXCR5 and a dead/live stain. Samples were subsequently analyzed by FACS. The gating strategy to identify the different follicular T-cell populations is partially illustrated and was performed as in [30]. Dot plots depicting the percentage of the tonsillar Tfh subsets of selected individuals, from the youngest (top) and oldest (bottom) groups of patients analyzed. Percentages and colors designate frequencies of the populations indicated. (**B**) Histograms presenting the mean percentage ± SD of the Tfh_GC_ and Tfh_M_ population frequencies determined as in A), from 55 individuals, distributed according their age. *p* value was calculated through unpaired *t* test, *p < 0.05; **p < 0.01

Tfh cells are the primary drivers of T cell–dependent GC responses. To extend our findings, we also assessed tonsillar T CD4^+^ cell populations. The Tfh cells locating within the GC (Tfh_GC_) express high levels of CXCR5 and PD-1 (CXCR5^high^ PD-1^high^). There are also Tfh in secondary lymphoid organs which express intermediate levels (CXCR5^int^ PD-1^int^) of those markers and localize in the mantle zone of the follicle (Tfh_M_) (Fig. 3A). The proportion of the latter steadily increased with increasing age (Fig. 3C). Conversely, the frequencies of Tfh_GC_ cells were significantly lower in children over 9 years old when compared with younger children, consistent with the decay of the proportion of B_GC_ cells population (Fig. 3B). In Table 3, the demographic data of analyzed patients for this assay.

**Table 3:** Basic demographic data of tonsils donors for Thf analysis

Age group (years)	Gender				Total	
	Males		Females			
	No^a^	%^b^	No^a^	%^b^	No^a^	%^b^
2–4	10	18,2	5	9,1	15	27,3
5–8	8	14,5	11	20	19	34,5
9–11	7	12,7	4	7,3	11	20
12–18	8	14,5	2	3,6	10	18,2
TOTAL	33	60	22	40	55	100

^a^The values represent the number of patients in each group who donated samples after undergoing surgery.

^b^The percentages were calculated relative to the total number of patients (*n* = 55)

Since we mostly work with a paediatric otolaryngology service, we usually receive samples up to 18 years old. We have previously shown that within such a cohort, the frequency of tonsillectomies peaked at 5–6 years old [4]. We further confirmed the data in the present manuscript. In Fig. 1B we present data from 76 samples, 41% of those fall in a single interval of age, the one including tonsils from children ranging from 5 to 8 years old. Children younger than 5 years old account for another 26% of the samples. Therefore, puberty specimens represent only a third of our samples. Moreover, samples from children over 12, represented 12% of the samples analyzed (Table 2).

Notably, while TH is the most frequent cause of tonsillectomy in children younger than 10, abscesses and RT are responsible for most tonsillectomies in teenagers [3, 9]. Our observations provide a putative mechanistic foundation to the temporal pattern reported for the causes of tonsillectomy, suggesting that the decrease in GC proportion holds critical consequences for some individuals around or after puberty.

### Decreased tonsillar GC reactivity after puberty

**Fig. 4:**
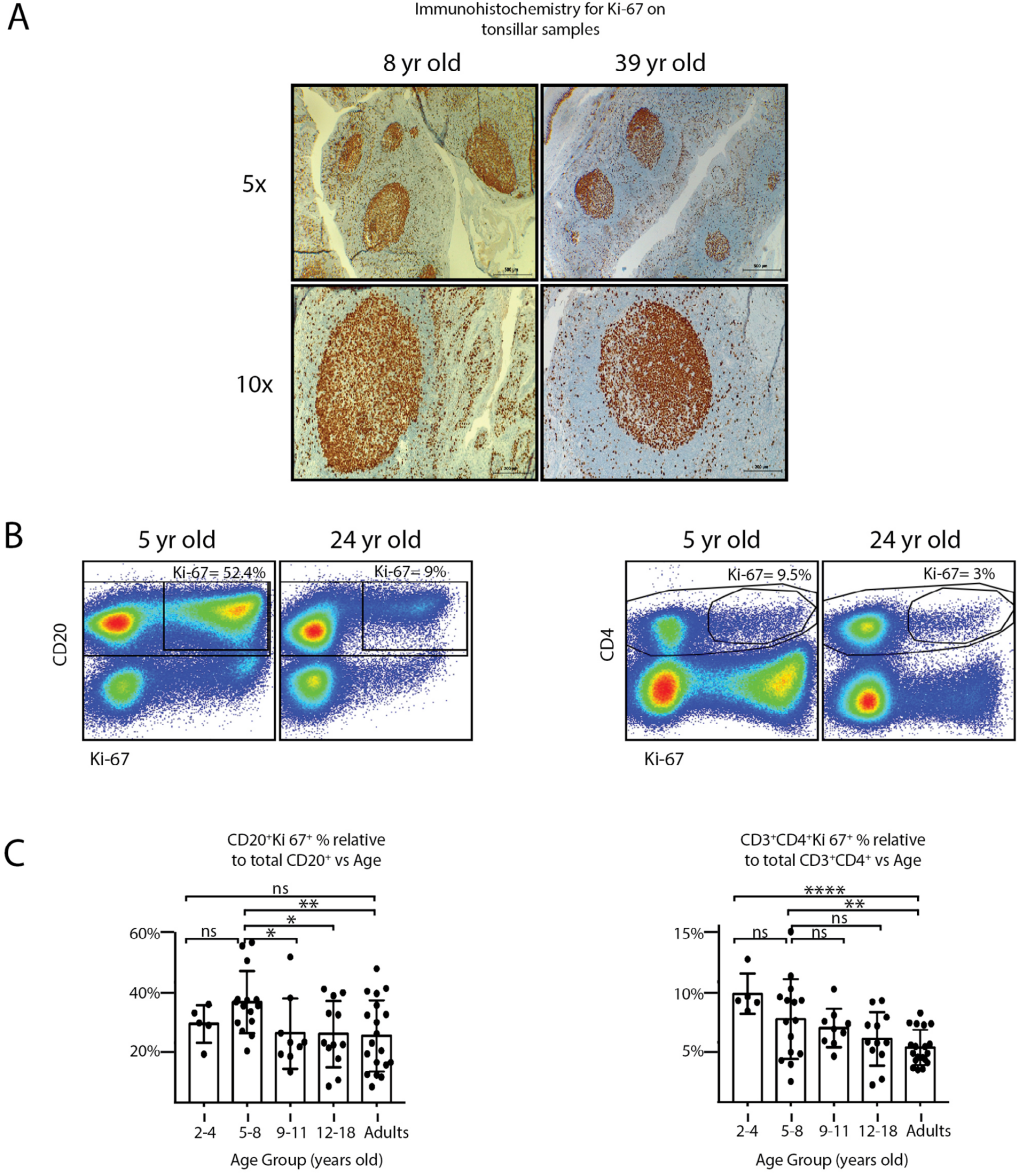
Teenagers and adults’ tonsils comprise less proliferative cells than children. (**A**) Representative immunohistochemistry staining of Ki-67 on tonsils biopsies from donors of the indicated ages at two different magnifications (also indicated). Scale bar, 500 and 200 μm, respectively. (**B**) Fresh TMC were stained for surface CD4, CD20 and CD3, also for intra-nuclear Ki-67 and a fixable viability dye. Samples were subsequently analyzed by FACS. The gating strategy to identify the proliferating B and CD4^+^ T cell populations is partially illustrated. Singlets were gated by plotting FSC-H vs. FSC-A for each sample (not shown). Within the singlets population, dead cells were determined by the fixable viability dye (not shown). Within the viable gate, lymphoid gate was determined through SSC-A vs. FSC-A (not shown). Left panels: dot plots depicting the percentage of the tonsillar CD20^+^Ki-67^+^ subsets from single donors of the indicated ages. Right panels: dot plots depicting the percentage of the tonsillar CD3^+^CD4^+^Ki-67^+^ subsets from single of the indicated ages. Percentages designate frequencies of the populations indicated. (**C**) Histograms presenting the mean percentage ± SD of the cell population frequencies determined as in B) from 60 individuals distributed according their age. *p* value was calculated through unpaired *t* test, *p < 0.05; **p < 0.01 and **** p < 0.0001

GC develop from the clonal expansion of replicating B cells. Hence, the persistence of GC responses is influenced by the proliferative capacity of the B cells. Ki-67 antigen expression is strictly associated with cell proliferation as it is expressed in the nucleus of dividing cells but not during the G0 phase. It is known that B_GC_ cells and plasmablasts exhibit the highest levels of Ki-67 expression of all tonsillar lymphocytes [10], being B_GC_ cells a much larger population than plasmablast at all ages (Fig. 4A). Importantly, epithelial cells which also express Ki-67, are not constituents of the fraction we work with (TMC).

In order to confirm the progressive loss of competence of the GC with the age of the patients, Ki-67 was used to identify and relatively quantify the proliferating cell populations at the time of the surgery, that is within fresh TMC (Table 4). We were able to incorporate adult samples for the assay (tonsils from people in their second and third decades of life). We detected proliferating cells in the B and T cell compartments which evidenced immune activity in all samples tested (Fig. 4A, B and C). As expected, the B cell compartment exhibited the highest proportion of proliferating cells driven by B_GC_ cells (Fig. 4A, B and C). There was a significantly higher percentage of CD20^+^Ki-67^+^ cells in children from 5 up to 8 years old (N = 15, 33.4% ± SD 10.4%) than that of older children (9–11 years old, N = 9, 23.0% ± SD 11.7%), teenagers (12–18 years old, N = 12, 23.0% ± SD 11.7%) and adults (N = 19, 22.8% SD 10.9%) (Fig. 4C). The toddlers (2–4 years old) presented no significant differences with any of the other groups (N = 5, 26.2% SD 6.2%). Of note, we analyzed few samples for Ki-67 staining from the toddlers group (N = 5).

**Table 4:** Basic demographic data of tonsils donors for Ki-67 analysis

Age group (years)	Gender				Total	
	Males		Females			
	No^a^	%^b^	No^a^	%^b^	No^a^	%^b^
2–9	1	1,66	4	6,66	5	8,33
5–8	6	10	9	15	15	25
9–11	6	10	3	5	9	15
12–18	8	13,33	4	6,66	12	20
Adultos (> 18)	13	21,66	6	10	19	31,66
TOTAL	34	56,66	26	43,33	60	100

^a^The values represent the number of patients in each group who donated samples after undergoing surgery.

^b^The percentages were calculated relative to the total number of patients (n = 60)

When scoring the proliferative capacity of CD4^+^ T cell populations, we found that the toddlers and the 5 to 8 years old group presented statistically significant higher frequencies of CD4^+^Ki-67^+^ (N = 5, 9% ± SD 1.7%) and (N = 15, 6.9% ± SD 3.3%) respectively, than adults (N = 19, 4.6% ± SD 1.5%) (Fig. 4C). Finally, we found that teenagers and adults exhibited very similar parameters, confirming the notion that tonsillar functional decay initiates around 10 years old [11], at least for the parameters that we analyzed.

These findings further validate the decline of tonsillar effector immune responses over time, which become relevant around the early onset of puberty. Taken all together, we decided to work with the samples divided in two groups, taking 10 years old as the dividing time point (in light of our own data and also in agreement with [3] and [11]).

### Co-expression of CD39 and CD73 membrane proteins in freshly isolated tonsillar B cells increases with age

**Fig. 5:**
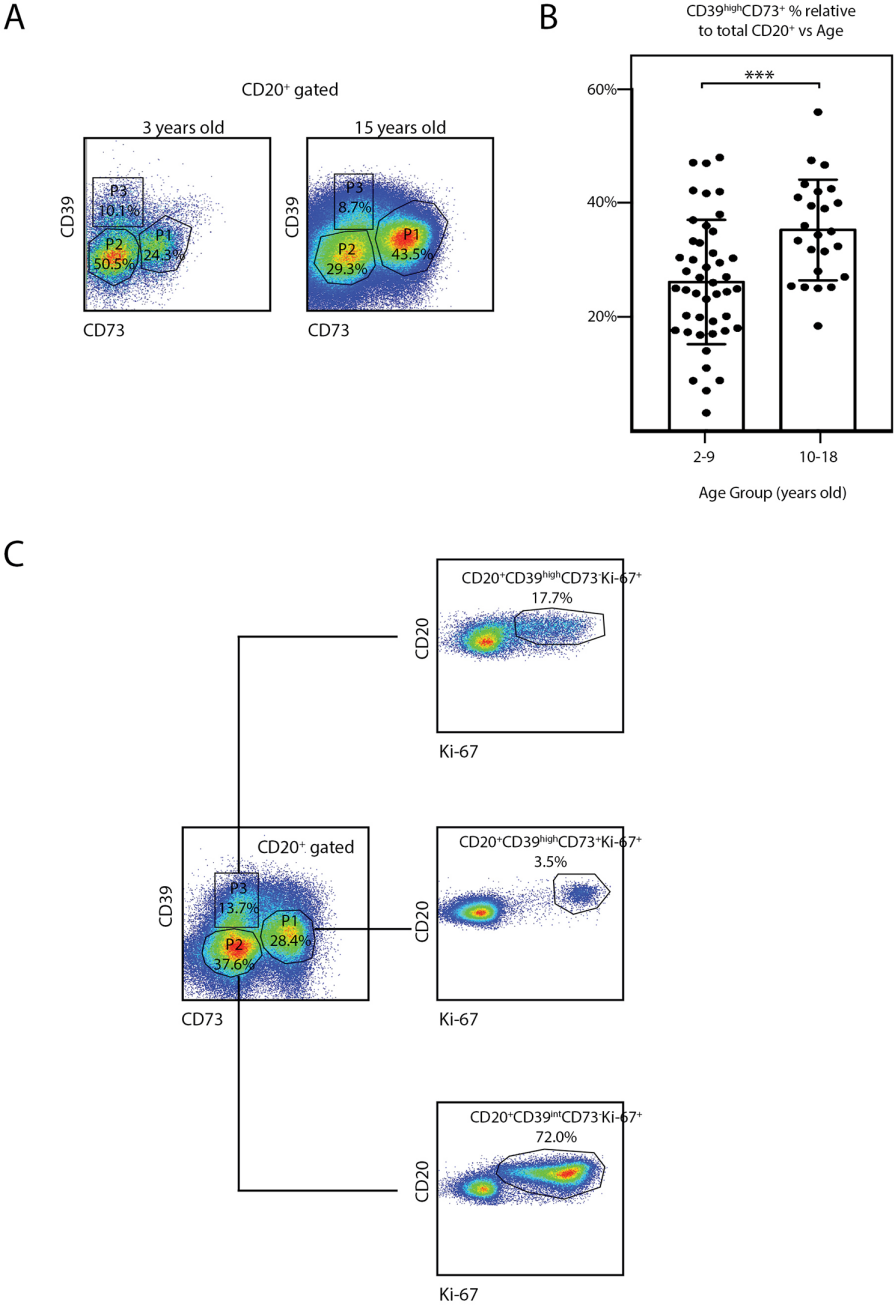
B-cells metabolically adapted to catabolize ATP increase with increasing age. (**A**) Fresh TMC were stained for surface CD20, CD3, CD73, CD39 and a dead/live stain. Samples were subsequently analyzed by FACS. The gating strategy to identify the B-cell population analyzed, is partially illustrated. Singlets were gated by plotting FSC-H vs. FSC-A for each sample (not shown). Within the singlets population, dead cells were determined by a viability dye (not shown). Within the viable gate, lymphoid gate was determined through SSC-A vs. FSC-A as well as the CD20 gate (not shown). Dot plots depicting the percentage of the tonsillar B cells co-expressing CD39^high^ and CD73 (P1), CD20^+^CD39^Int^CD73^−^ (P2) and CD20^+^CD39^high^CD73^−^ (P3) populations of selected individuals, from the children (left) and teenagers group (right). (**B**) Histograms presenting the mean percentage ± SD of the CD20^+^CD39^high^CD73^+^ population frequencies determined as in A), from 69 individuals, distributed according their age. *p* value was calculated through unpaired *t* test, ***p < 0.005. Frequencies of the P2 and P3 cell populations were not recorded for this paper. (**C**) Fresh TMC were stained for surface CD20, CD4, CD8, CD73, CD39 and intra-nuclear Ki-67. Middle left hand panel: analysis was performed as in A), the P1, P2 and P3 cell populations from a donor are depicted. Right hand panels: representative dot plots of proliferating cell populations within the P1, P2 and P3 gates as indicated by the respective lines. Percentages designate frequencies of the populations indicated relative to their respective gate. Gates were manually adjusted due to the changes experienced by the cells in culture upon each treatment

As the patients approach youth, a number of biological factors could be related to the deterioration of their tonsillar effector immune responses. We have previously shown that hyperplastic tonsils from young children endure bacterial growth able to breach the epithelial barrier [4]. Such damage to epithelial tissues might trigger tolerance programs intended to restrain immunopathology, assisting the transition of tissues from pro-inflammatory to anti-inflammatory conditions [12, 13]. To investigate a potential metabolic adaptation of B cells promoting a suppressive behavior upon years of hyperplasia and chronic inflammation, we compared co-expression of CD73 and high levels of CD39 on CD20^+^ cells (Fig. 5A) from TMC of children younger and older than 10 years old (Table 5). We found that samples from patients over 10 years old presented a statistically significant higher CD20^+^CD39^high^CD73^+^ cell population than those from younger children (Fig. 5B and 35.3% ± SD 8.9% vs. 26.1% ± SD 10.9% respectively, *n* = 69, *p* < 0.005). Thus, we confirmed that the declining of GC reaction with ageing and chronic inflammation, correlated with an increment in the proportion of a B cell phenotype associated with immunosuppressive activity. Moreover, we found that the majority of CD20^+^CD39^high^CD73^+^ cells were in G0 phase as evidenced by a negligible percentage of CD20^+^CD39^high^CD73^+^ Ki-67^+^ population within the subset. Notably, the majority of the proliferating cells appeared in the CD20^+^CD39^Int^CD73^−^ cell fraction (Fig. 5C), which is significantly higher in samples of younger ages than older ones, as expected in light of the results shown in the previous sections.

**Table 5:** Basic demographic data of tonsils donors for CD39 and CD73 analysis

Age group (years)	Gender				Total	
	Males		Females			
	No^a^	%^b^	No^a^	%^b^	No^a^	%^b^
2–9	29	42	19	27,5	48	69,6
10–18	14	20,3	7	10,1	21	30,4
TOTAL	43	62,3	26	27,6	69	100

^a^The values represent the number of patients in each group who donated samples after undergoing surgery.

^b^The percentages were calculated relative to the total number of patients (*n* = 69)

Collectively, these results indicate a strong correlation between the appearance of RT as the main cause of surgery in teenagers and the boost of immune regulation of GC reactions with increasing age.

### In vitro proliferative capability of B cells isolated from tonsils

**Fig. 6:**
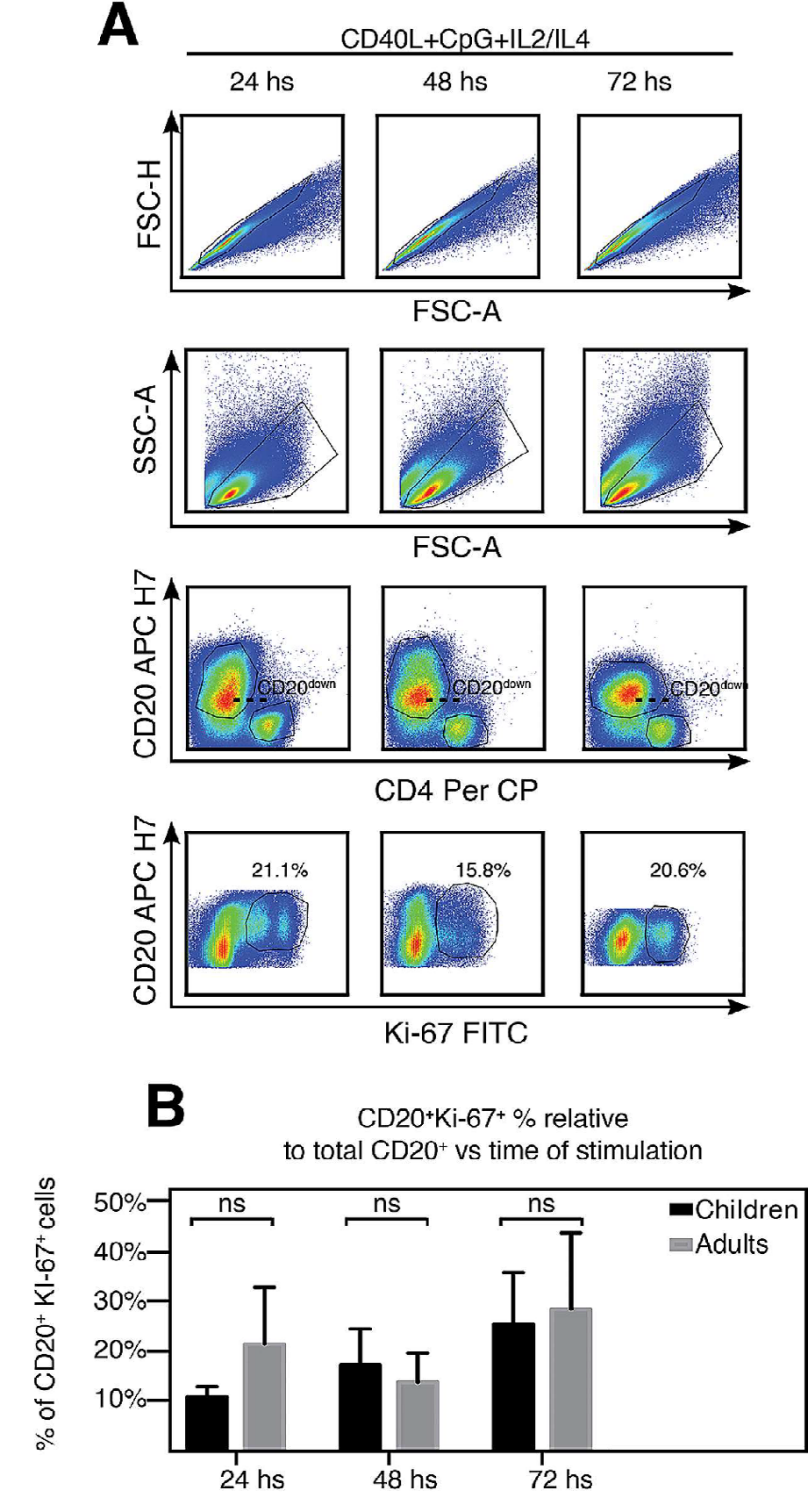
Proliferative response of tonsillar B-cells from donors at different ages. (**A**) Freshly isolated TMC were cultured on CpG + CD40L + IL2 + IL4, for the time points indicated on the top of each panel. Cells were stained for surface CD20, CD4, CD8, CD73, CD39 and intra-nuclear Ki-67. Samples were subsequently analyzed by FACS. Gating strategy is illustrated. Singlets were gated by plotting FSC-H vs. FSC-A for each sample (upper panels). Within the singlets population, apoptotic and viable cells were distinguished by differences in forward and side scatter. Such scatter-based assay has good correspondence with results obtained by fluorescein isothiocyanate (FITC)- annexin staining for distinguishing the viability of untransformed human B cells [31, 32]. Within the viable gate, B cells were identified through the CD20 vs. CD4 dot plots. Dashed lines show the decline of expression of CD20 post stimulation. Proliferating B cells were recorded as the CD20^+^CD4^−^Ki-67^+^ cell population of the bottom dot plot panels and percentages designate the respective frequencies at each time point. Data from one donor representative of the 8 patients whose TMC were cultured. Gates were manually adjusted due to the changes experienced by the cells in culture upon each treatment. (**B**) Histograms presenting the mean percentage ± SD of the CD20^+^CD4^−^Ki-67^+^ population frequencies determined as in A), from 8 individuals, distributed according their age (4 children and 4 teenagers and adults). *p* value was calculated through unpaired *t* test, ns = con significant differences

The phenotypic analyses we have shown so far provide a snapshot of the tonsillar lymphocytes at the time of the surgery. Furthermore, in the previous section we suggested that a chronically disturbed microenvironment might trigger metabolic changes in the tonsillar B cells. Hence, it was of interest to investigate the intrinsic lymphoid ability to functionally respond to stimulation ex vivo, independently of the histological microenvironment. To address this issue, we cultured TMC from patients at different stages of life and evaluated Ki-67 expression at 24, 48 and 72 h (Table 6). These cultures were supplemented with the toll-like receptor 9 agonist CpG and CD40L which target B cells as well as IL4 and IL2 which promote primary B and T cell survival. We have long used this system to study different aspects of the TMC functional response in adults [14], children [4] and both [2]. Briefly, stimulation is associated to elevated levels of cell death since the cultures are initially dominated by a variety of terminally differentiated and highly activated B cells [4, 14]. In fact, we routinely use this decay in the viability of the TMC cultures as well as the CD20 down-modulation [4, 15] to monitor for suitable activation (Fig. 6A). We tracked Ki-67 levels over time in TMC cultures of 8 different patients at different stages of life. We included 4 individuals in the children´s group (3, 5, 6 and 6 years old) and 4 individuals in the post-puberty group (11, 12, 15 and 32 years old). All cultures exhibited a similar pattern of Ki-67 expression, with no significant differences between them. We did not detect significant differences in the percentages of CD20^+^Ki-67^+^ at any time point (Fig. 6B). Such results are in agreement with our own reports regarding the fitness of TMC in culture in general, independently of the age of the samples [2, 4, 14] and with those from other researchers in a different model [16]. Importantly, we have not tested samples older than 39 years old. In all cases, we observed a down-regulation of CD73 expression in cultivated CD20^+^ cells at 24 hs of culture which ended up in a complete absence of a CD20^+^CD73^+^ population by the second day of culture (Suppl Fig. 2). There was a slight shift on the fluorescence of the CD20^+^CD73^−^ population that resulted from the increment in the auto-fluorescence of the stimulated TMC confirmed by comparison with FMO controls and in agreement with previous reports [17]. The analysis of CD39 expression in culture was complex as its resting expression was already more complex than that of CD73 (Fig. 5). A thorough study on the function of both markers on tonsillar B cells is beyond the scope of the present manuscript and will be discussed elsewhere (Pastor et al. manuscript in preparation). Taken the proliferation results and the plasticity in the expression of the immune check points together, we concluded that within the range of samples we have worked with, the decline in the robustness of the tonsillar GC response with age would not be attributable to exhausted B and T cell compartments as cultured TMC from all patients exhibited a typical B cell response, disregarding their starting proliferative status.

**Table 6:** Basic demographic data of tonsils donors for Ki-67 kinetics analysis

Age group (years)	Gender				Total	
	Males		Females			
	No^a^	%^b^	No^a^	%^b^	No^a^	%^b^
Children	1	12,5	3	37,5	4	50
Teen an adults	3	37,5	1	12,5	4	50
TOTAL	4	50	4	50	8	100

^a^The values represent the number of patients in each group who donated samples after undergoing surgery.

^b^The percentages were calculated relative to the total number of patients (*n* = 8)

## Discussion

Human tonsils are considered comparable to the nasopharynx-associated lymphoid tissue (NALT) of rodents, they are part of the mucosa-associated lymphoid tissue (MALT). They also show similarities with lymph nodes and participate as effector organs of local systemic immunity as well, which make them rather unique lymphoid structures. Here, we have used them as a model to study the impact of chronic inflammation and time on B cell mediated immunity, in particular.

We found that tonsils from young children (before puberty) yielded a significantly higher proportion of GC cell populations (B_GC_ cells and Tfh_GC_ cells) than samples from teenagers and adults. Moreover, younger GC cells presented higher proportion of proliferative immune cells than those from older ones. In contrast, the older children presented higher percentage of Ag-experienced B cells, like memory B cells. Such observations are in line with previous studies on B and T cells in a number of lymphoid compartments, as people age [10, 18]. We have previously demonstrated that adults’ circulating and tonsillar memory B cells represent different memory B cell pools with different functional responses [14]. The distinction between re-circulating and tissue resident memory B cells is currently well established also by other authors, in mice and in other human tissues like lungs and gut [19–21]. In spite of such advances, we have not phenotypic markers able to discriminate circulating and resident tonsillar memory B cells yet. Further work into assessing the specificity of those memory B cells is required to elucidate whether the accumulation is led by tonsillar resident memory B cells or not.

The depreciation of the tonsillar immune effector mechanisms with advancing age detected by phenotyping correlates with a shift in the causes of surgery. Abscesses and chronic or recurrent tonsillitis are the main grounds for tonsillectomy after puberty [3], which evidence the failure of the local immune system to restrain infections and their systemic manifestations, in teenagers and young adults.

Bacterial and viral infections are thought to be a major source of tonsillar disease pathogenesis leading to surgery. ADO is a purine nucleoside associated with various immune-pathological processes [22]. In this paper we also demonstrate that CD20^+^CD39^high^CD73^+^ cell population expands post puberty. CD39 and CD73 are two ecto-nucleotidases that act sequentially to catabolize ATP to ADO. This biochemical pathway conducing to ADO generation is very active in inflammatory microenvironments. The immunological outcome of ADO production is an immunosuppressive microenvironment [23]. This is not unexpected upon years of constant inflammation, as organisms have evolved mechanisms to regulate immunity to keep the physiological function of the tissue in the context of the persistent presence of an insult, as it is the case of the ENT patients. In the future, it would be interesting to investigate actual ADO levels produced by tonsillar CD20^+^CD39^high^CD73^+^ of patients at different stages of their lives.

We have previously reported on the higher proportion of Bregs as defined by IL10 secretion [24] and GC declining when comparing samples from RT and TH [2]. Therefore, we have extended those findings here, by monitoring an alternative Breg population and also by taking in account the effect of the age of the samples. Of note, the age associated observations described here occurred in all samples irrespective of the bacterial species that they harbored, which were numerous and were all considered normal oropharyngeal commensals (data not shown).

A point of particular interest to discuss is whether the variations in the lymphocyte subsets and the consequent shift from a pro-inflammatory microenvironment to a more suppressive one, can be extrapolated to people that do not present any disease that would justify tonsillectomy. In a prospective family-based cohort study, 16% of all the adults recruited reported an illness with sore throat and fever over a 1-year time frame. Such fraction scales up to 47% in teenagers from 12 to 18 years old, and to 40%, in children below 12 years old [25]. Hence, teenagers and children in general seem to be much more vulnerable to tonsillitis caused by the most usual pathogens, than adults. The accumulation of B cell memory population with age that we observed in the tonsillar samples would account for the lower incidence in adulthood. On the other hand, while a predominant and consolidated memory B cell pool should be protective on re-exposure to the typical pathogens, it would leave adults more vulnerable to upper respiratory infections and tonsillitis from novel pathogens, than children. Interestingly, that was the case when COVID-19 emerged [26]. Of note, tonsils were identified as crucial sites of SARS-CoV-2 infection in children for an undetermined prolonged time, and surprisingly, with no symptoms [27].

The persistent sub-clinical infection underlying the hypertrophy of the samples from young children as well as the manifested recurrent infections present in older donors, both should have an impact on the tonsillar immune landscape, contributing to precipitate the effects of hormones, genetics, habits, and time in the samples we work with. Concerning the latter, dampened GC reactions are a trait of ageing that is conserved across species [28]. Interestingly, we found that when isolated from their histologic microenvironment, stimulated tonsillar B cells proliferated ex vivo to a similar extent at all ages tested. This is not an unexpected observation as the GC reaction needs the interplay of a number of cell types which must be organized in space and time as well as the appropriate levels of antigenic stimulus. All these factors are affected by chronic inflammation and ageing processes. For instance, it has long been established that the main routes of tonsillar Ag uptake are reduced in adults [29]. Anatomical milieu is a crucial aspect of the immune response and changes in the tonsillar stromal architecture affect the coordination of cellular and molecular interactions that tune local B cell responses. In this regard, we have already speculated on the influence of a long lasting inflammatory microenvironment in the local accumulation of B cells expressing ecto-nucleotidases which enable ADO production. Interestingly, when we isolated TMC from their histological context and stimulated them ex vivo, the expression of ecto-nucleotidases in B cells was downregulated at a similar extent in samples of different ages.

## Conclusions

Our data supports the notion of a link between the age-dependent causes of tonsillectomy and a reduction in the proportion of effector cells (B_GC_), an increase in the proportion of memory B cells and an increase in the proportion of B cells with regulatory function as the patients age. With regards to T cells, Thf_GC_ decrease and Thf_M_ increase as age increases in the cohort of patients that we analyzed. Moreover, the proliferative status of local CD4^+^ T cells resulted as affected as the B cell compartment.

The findings described here are limited to tonsils that needed to be excised at different stages of life. We have discussed in a paragraph of the previous section the rationale to hypothesize whether healthy tonsils could undergo similar immunological changes when ageing as the ones we have just shown.

Overall, the study contributes to the comprehension of the changes in the tonsillar immune response when advancing life stages for some people and could eventually help to predict the different results, in terms of immune responses, to local antigenic challenges in the life span of individuals.

## Material and methods

**Study population.** This study recruited 95 surgical patients aged 2-39 years old from 2 different institutions in total (Suppl. Fig. 1 and Table 1 for gender and age distribution). Patients below 18 years old (children and teenagers, N=76) were enrolled from the Clinical Hospital ‘José de San Martín’. Adult patients (N=19) were enlisted from the Institute of Otolaryngology Arauz. We excluded samples from patients with any kind of immunodeficiency, neither primary nor secondary. We also excluded any patient taking medication a month prior the surgery (antibiotics, corticoids, etc).

Due to a number of reasons, we were not able to perform the whole set of determinations described in the previous sections to all the samples (Suppl. Fig. 1). We detailed in the corresponding figure legend the precise number of samples used in the experiments shown in each of those figures. Adults were recruited later in the study than children and teenagers.

**Isolation of cells.** Primary human mononuclear cells were isolated from tonsils obtained from patients undergoing tonsillectomy. The particular number of samples per experiment were detailed in the corresponding Figure legends. TMC were prepared as follows. Briefly, tonsils were collected in phosphate buffered saline (PBS) buffer containing 50 µg ml^− 1^ amphotericin B (Richet, BA, Arg). Tissues were chopped with a scalpel and passed through a 70 μm-pore-size cell strainer (Falcon, Thermo Fisher, BA, Arg). TMC were purified by density gradient centrifugation with Ficoll-Hypaque (GE Healthcare, Uppsala, Sweden). The viability of primary cells, as determined by trypan blue exclusion was greater than 99% in all preparations. Informed consent was obtained from subjects before the study. The institutional ethics committee (Clinical Hospital, School of Medicine, Buenos Aires and Institute of Otolaryngology Arauz, Buenos Aires) approved the collection and use of clinical material, conformed to the provisions of the Declaration of Helsinki (as revised in Edinburgh 2000). Informed consent was obtained from all participants and/or their legal guardian/s. FACS experiments were performed with freshly isolated cells and cultured cells.

**Cell culture.** TMC were cultured in IMDM medium (Life Technologies, CA, USA) containing 10% heat-inactivated fetal calf serum, 2mM L-glutamine, 100 U/ml penicillin, 100 µg/ml streptomycin, 20 mM 4-(2-hydroxyethyl)-1-piperazineethanesulfonic acid buffer (HEPES), 1 mM sodium pyruvate and 50 µM 2-mercaptoethanol (all from Invitrogen, CA, USA). Human IL2 (20 ng/ml; R&D Systems, MN, USA) and human IL4 (20 ng/ml; R&D Systems, MN, USA) were added immediately before experiments also as supplements. When indicated, human recombinant CD40L (250 ng/ml; R&D Systems, MN, USA) and 25 µM CpG-ODN 2006 (InvivoGen, CA, USA) were used. Cells were cultured at 1 × 10^6^ cells/ml either in 24-well culture plates (1 ml) or 48-well culture plates (0.5 ml).

**Antibodies and fluorescence-activated cell sorting (FACS).** Fluorochrome conjugated mAbs specific for human CD3 (Pacific Blue, clone SK7, BioLegend), human CD20 (FITC, clone L27 and APC H7 clone 2H7), human CD4 (PerCP, clone SK3, BioLegend), CD8 (APC Cy7 clone SK1, BioLegend), CD39 (APC, clone TU66, BD Pharmingen), CD73 (PE, clone AD2, BD Pharmingen), CD27 (FITC, clone M-T271, BD Pharmingen), CD38 (APC, clone HIT2, BD Pharmingen), CD44 (Bv510, clone IM7, BioLegend), CXCR5 (AF488 clone RF8B2, BD Pharmingen), PD1 (Bv711, clone EH12.2H7, BioLegend), CD10 (PE, clone ALB1, Beckman Coulter), Ki67 (FITC, clone B56 RUO, BD Pharmingen) and respective isotype control mAbs were purchased from BD Biosciences (CA, USA) and Biolegend (CA, USA).

To detect Ki-67 transcription factor in the cells, the latter were incubated with Fixation/Permeabilization (eBioscience FOXP3/Transcription, Invitrogen) for 45 min and washed with Permeabilization Buffer (eBioscience FOXP3/Transcription, Invitrogen). Then, the cells were stained with anti-Ki-67 mAb.

Cells were acquired using FACSAria II (BD Biosciences, CA, USA) and analyzed with FlowJo software (Treestar, OR, USA). Single stained controls were used to set compensation parameters. Fluorescence minus one and isotype-matched Ab controls were used to set analysis gates.

**Immunohistochemistry.** The 5 μm tissue sections mounted on silanized glass slides were deparaffinizated by two consecutive 5 min incubations xylene each, hydrated in decreasing concentrations of alcohol (100%, 96% and 70%) for 5 min each, followed by antigenic unmasking with Sodium Citrate Buffer (0,05 M, pH 6.0) in a thermostatic water bath at 95 °C for 45 min.

The endogenous peroxidase was blocked by incubating tissue sections with 35% H_2_O_2_, 98.8% methanol PA (Synth) diluted in PBS pH 7.6 for 30 min, then washed with PBS-1X pH 7.6. The nonspecific binding sites were blocked with Bovine Serum Albumin (BSA) 5%. Ki-67 + cells were detected by incubating with the primary antibody. The antibody dilution was 1/100 Ki-67 (Rabbit monoclonal clone SP6, TecnoLab, 275R-15). Slides were incubated one hour at 4 °C and washed three times with PBS 1X, pH 7.6.

For the immunohistochemical staining system and counterstaining with hematoxylin, a fully automatic staining device, the Ventana BenchMark XT (Ventana Medical Systems, Roche Diagnostics Division) was used. The device uses a biotin-free, HRP multimer-based hydrogen peroxide substrate and 3, 3’-diaminobenzidine tetrahydrochloride (DAB) chromogen (UltraVIEW Universal DAB Detection, Catalog number 760 − 500, Ventana Medical Systems, Tucson, USA).

The slides were examined using the Leica DM500 optical microscope (Leica Camera, Wetzlar, Germany) at magnification of 20x and 40x.

**Statistics.** The results were analyzed using GraphPad Prism 7.0 and GraphPad Prism 8.0 software. The normality of variable distribution was assessed by the Shapiro-Wilk test. The statistical analysis of the results was performed using the unpaired t test, and a p value of < 0.05 was considered significant unless indicated otherwise.

### Electronic supplementary material

Below is the link to the electronic supplementary material.

**Supplementary Material 1: Supplementary Figure 1.** Study flow chart

**Supplementary Material 2: Supplementary Figure 2.** CD73 expression by cultured tonsillar B cells. Freshly isolated TMC were cultured on CpG+CD40L+IL2+IL4, for the time points indicated. Cells were stained for surface CD20, CD4, CD8, CD73, CD39 and intra-nuclear Ki 67. Samples were subsequently analyzed by FACS. Gating strategy is illustrated in Figure 6. Histograms for CD73 fluorescence from CD20+ cells over 3 days displayed as half offset graphs

## Data Availability

The authors confirm that the data supporting the findings of this study are available within the article. In case of need of further details, Eloísa Arana would provide them upon reasonable request.
