# Supplementary figures and images for: Role of germinal center and CD39highCD73+ B cells in the age-related tonsillar involution
Source: Immun Ageing. 2024 Apr 12;21:24. doi: 10.1186/s12979-024-00425-4 (PMC11010345; doi:10.1186/s12979-024-00425-4)

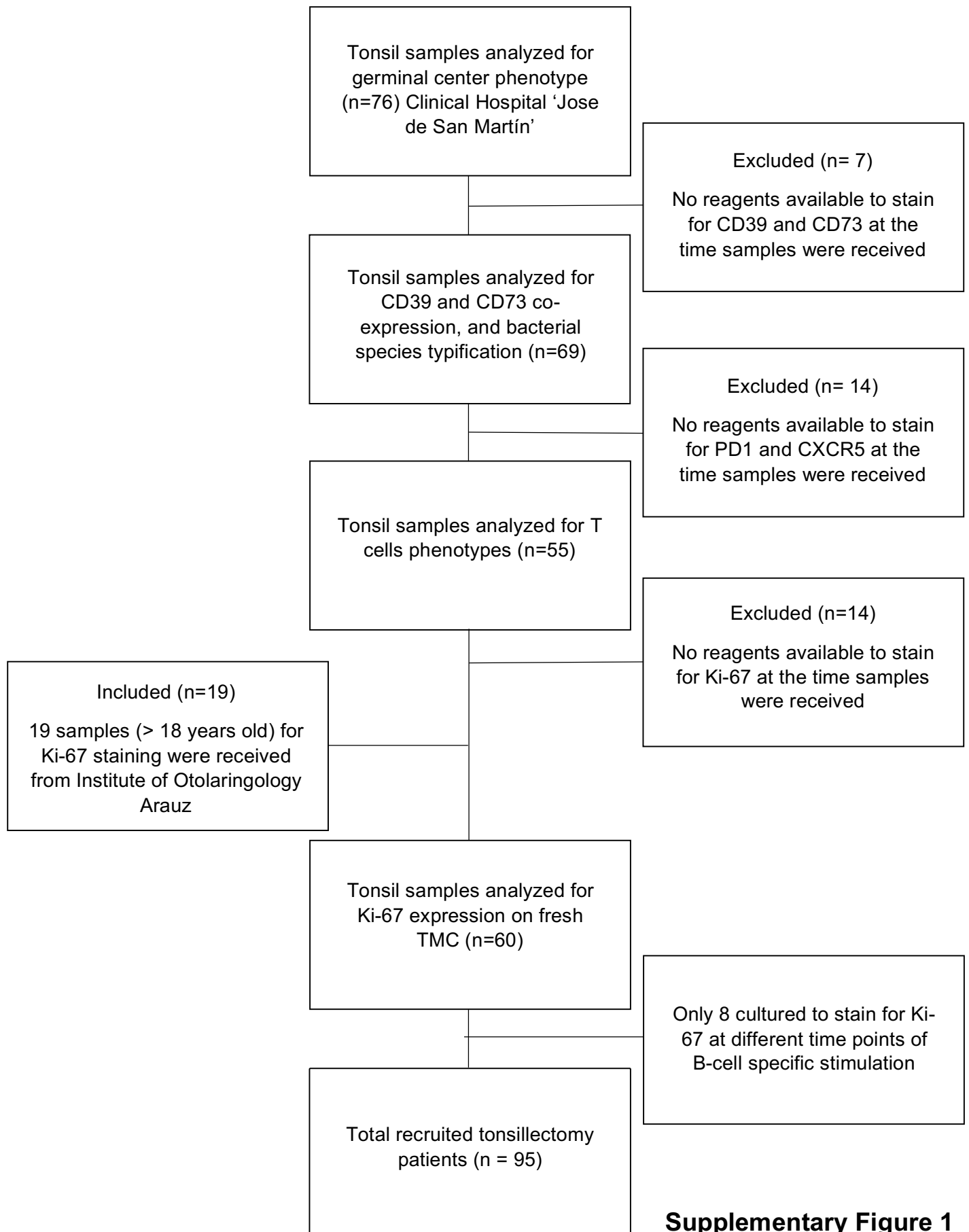

**Supplementary Figure 1**

Supplement: Supplementary file 1 — Supplementary Material 1: Supplementary Figure 1. Study flow chart [file 12979_2024_425_MOESM1_ESM.pdf]

CD73 expression on B cells at different time  
points of cultures

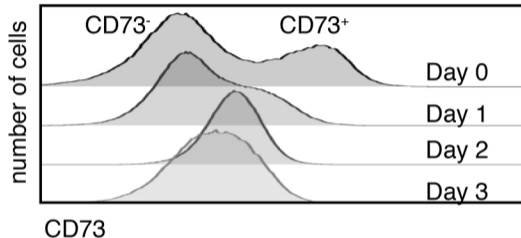

**Supplementary Figure 2**

Supplement: Supplementary file 2 — Supplementary Material 2: Supplementary Figure 2. CD73 expression by cultured tonsillar B cells. Freshly isolated TMC were cultured on CpG+CD40L+IL2+IL4, for the time points indicated. Cells were stained for surface CD20, CD4, CD8, CD73, CD39 and intra-nuclear Ki 67. Samples were subsequently analyzed by FACS. Gating strategy is illustrated in Figure 6. Histograms for CD73 fluorescence from CD20+ cells over 3 days displayed as half offset graphs [file 12979_2024_425_MOESM2_ESM.pdf]
